# The long shadow of accumulating adverse childhood experiences on mental health in the United Arab Emirates: implications for policy and practice

**DOI:** 10.3389/fpubh.2024.1397012

**Published:** 2024-07-19

**Authors:** Anthony Murphy, Dawn England, Iffat Elbarazi, Neal Horen, Toby Long, Zeina Ismail-Allouche, Cairo Arafat

**Affiliations:** ^1^School of Psychology, The University of Birmingham, Edgbaston, United Kingdom; ^2^School of Education, The University of Birmingham, Edgbaston, United Kingdom; ^3^College of Medicine and Health Sciences, United Arab Emirates University, Al Ain, United Arab Emirates; ^4^Center for Child and Human Development, Georgetown University, Washington, DC, United States; ^5^Centre for the Study of Learning and Performance, Concordia University, Montreal, QC, Canada; ^6^Early Childhood Authority, Abu Dhabi, United Arab Emirates

**Keywords:** child abuse, child neglect, adverse childhood experiences, United Arab Emirates, adult outcomes

## Abstract

**Introduction:**

This study investigates the cumulative effects of adverse childhood experiences (ACEs) on adult depression, anxiety, and stress in Abu Dhabi, controlling for demographic factors, lifestyle, and known health and mental health diagnoses.

**Methods:**

Utilizing a cross-sectional design and self-report measures, the research aims to fill a critical gap in understanding the specific impacts of ACEs in the UAE. Based on a multi-site, cross-sectional community sample of 697 residents of Abu Dhabi.

**Results:**

The findings reveal significant variances in current screening values for depression, anxiety, and stress attributable to ACEs after controlling for demographic factors, lifestyle risk factors, and adult diagnoses of health and mental health conditions.

**Discussion:**

The results underline the lifelong impact of ACEs and reinforce the importance of early identification and intervention. In particular, the implications for policy and practice in understanding and mitigating ACEs long-term effects on mental health are considered.

## Introduction

Adverse childhood experiences (ACEs), encompassing various forms of abuse, neglect, and household dysfunction, have been recognized as significant predictors of mental health outcomes across the lifespan (1, 2). The ubiquity and severity of these experiences necessitate a comprehensive understanding of their long-term impacts, especially on depression, anxiety, and stress globally. While substantial research underscores the link between ACEs and poorer mental health outcomes, there remains a critical gap in understanding these relationships when accounting for demographic variables, lifestyle factors, medical history, and pre-existing mental health diagnoses (3).

The biological, psychological, and social implications of ACEs contribute to their enduring influence on mental wellbeing. Early adversity is thought to disrupt brain development, impacting stress response systems and emotional regulation (4, 5). These neurobiological changes make individuals more susceptible to experiencing and sustaining negative emotions such as depression, anxiety, and stress (6), alongside impaired coping mechanisms, negative self-perceptions, and social difficulties, further escalating vulnerability to mental health issues across the lifespan (7). This understanding and confirmed trajectory of risk underscores the need for comprehensive approaches to mitigate the impact of ACEs on mental and behavioral health, emphasizing early identification, linkage to care, and access to trauma-informed services and supports. This holistic approach is crucial for addressing the multifaceted nature of mental health outcomes associated with ACEs, taking into account the broader context of individuals’ lives including demographic characteristics and lifestyle factors (3).

Despite widespread recognition of the ACE-mental health link globally (8), research focusing on specific Arab populations remains limited. While studies in Middle Eastern countries (9–11) provide valuable insights, the unique socio-cultural and demographic characteristics of the United Arab Emirates are poorly represented in psychology and public health literature. This is particularly pressing given that the burden of non-communicable disease, BMI-related health concerns, and high rates of smoking are considerable public health concerns (12), cardiovascular disease accounts for a quarter of deaths (13), and up to 29% of the populations exhibit high levels of depression (13). As such, there is a clear need for a dedicated investigation into the extent and impact of ACEs. Initial efforts by Long et al. (14) map the extent and nature of ACEs in the UAE, as well as their predictive impact on diagnosed health, mental health, and risk-related behaviors. They identify that 65% of participants report at least one ACE, with a greater number of ACEs being present in female respondents, and that cumulative ACEs are predictive of a suite of physical health and mental health outcomes in adults. Critically, their findings rely on binary, present/absent accounts of all diagnoses, where challenges around reporting, due to stigmatization, contribute at least in part to underdiagnosis of mental health outcomes (15). It, therefore, stands to reason that screening values for psychological suffering among community members represent a key addition to this emerging literature by developing a richer understanding of the predictive factors on depression, anxiety, and stress, complementing existing work conducted.

Additional context emerges when one considers young people in the UAE who report experiencing emotional abuse and neglect. They often display significant signs of depression, reduced self-esteem, increased screen time, and higher instances of smoking and tobacco use (16, 17). Despite the UAE’s strong legal frameworks to encourage reporting of child abuse cases, it is estimated that up to 90% of Child Abuse and Neglect (CAN) incidents remain unreported, particularly within Arab families (17), highlighting the importance of self-reported measures to capture these issues. This underreporting is critical for understanding child welfare in the UAE, which faces unique challenges due to its relatively large youth population compared to other affluent nations (18). This demographic context underscores the need to understand the factors that contribute to the burden of disease attributable to ACEs, especially considering the rising burden of depression, anxiety, and stress (13), alongside the routine underdiagnosis of mental health needs in the Middle East (15).

This study aims to fill this crucial gap by exploring the cumulative effects of ACEs on adult depression, anxiety, and stress in a representative sample of adults in Abu Dhabi while controlling for demographic factors, lifestyle factors, and adult health and mental health diagnoses. Existing literature highlights these social determinants of health for inclusion in population-level ACE studies (19–21), supported by theoretical frameworks such as the Bio-ecological model of human development (22) and its application to ACEs and other traumas experienced within a certain cultural and social ecology (23). Given the established literature and theoretical foundations of levels of environmental influence, it is hypothesized that increases in reported ACEs will significantly contribute to explained variance in depression, anxiety, and stress screening values when controlling for confounding factors related to demographics, lifestyle factors, and adult health and mental health diagnoses. Confirming these factors would provide nuanced insight beyond the presence or absence of mental health diagnoses, contributing to the explained variance in current screened values of psychological suffering among members of the Abu Dhabi community. This differentiation is particularly important due to the acknowledged challenges in accurately assessing mental illnesses in the community in the UAE (24). The significance of this study extends beyond academia, offering insights for clinicians, policymakers, and educators on the importance of early identification and intervention in cases of childhood adversity. It also underscores a significant fiscal imperative to address this public health concern.

## Methods and materials

### Design

As part of a broader study into community-level ACEs, health, wellbeing, and behaviors, this cross-sectional study examines the retrospective ACEs and their impact on adult depression, anxiety, and stress, above and beyond the influence of demographic variables, lifestyle factors, and medical and mental health diagnoses. Importantly, this study uses self-reported measures and screening values across a large cross-section of the community rather than relying on formalized institutional data to assess occurrences and co-morbidities in the population where cultural and institutional barriers lead to significant under-reporting (15).

### Participants and procedure

Adults (i.e., 18 years and older) living in Abu Dhabi were considered for the sample (N = 697). The sample was relatively diverse, with 56% women and 34% Emirati nationals. When asked about marital status, 66% of the participants reported being married, 26% single, and 7% divorced or separated. 53% of the participants reported having a child. Regarding employment, 71% of the participants reported full-time employment, 23% part-time, 11% unemployed, and 4% self-employed. Finally, 23% of the participants reported postgraduate training, 54% attained university education, and 23% graduated high school or less.

The use of convenience sampling facilitated comprehensive engagement with Abu Dhabi’s communities, where the distribution of the questionnaire was executed through the Qualtrics online survey platform, reaching the wider community via professional connections, key informants, and collaborating organizations, as well as in-person distribution. Given the nearly universal (98%) social media usage in the UAE (25), a social media campaign was also employed. Surveys were carried out solely in Arabic and English—Arabic as the UAE’s mother tongue and English as its *lingua franca* in business. No incentives or compensation were offered for participation. Trained research assistants were available to perform face-to-face interview versions of the questions, at the request of participants. This was incorporated as a response to the sensitive nature of the ACE-IQ and based on stakeholder feedback relating to culture and sensitivity. The uptake of this option was zero.

Trained research assistants were available to perform face-to-face interviews at the request of participants. This was in place due to the sensitive nature of the ACE-IQ scale and was a response to stakeholder feedback relating to cultural sensitivity. Despite being made fully aware of this, no participants chose that option. Participants were informed of their rights and a rough estimate of the time taken to complete the survey and were given a suite of physical/mental health referral contacts in the event of participation leading to distress, prior to consenting. Participants were informed of their right to refrain from answering any question they were not comfortable answering and to withdraw without justification and were provided with a unique participant ID and given the right to remove their data within 2 weeks of data collection, though no participants withdrew. The research was approved by the Institutional Review Boards of *Omitted for peer-review* University Medical Center, the *Omitted for peer-review* University, and the Abu Dhabi Department of Health.

### Materials

#### Demographics

The survey incorporated a demographic section to gauge the characteristics of the participants, ensuring alignment with the specified criteria for inclusion and exclusion. Information on participants’ age, gender, nationality, residency, educational background, marital status, parenthood, and job status was gathered, with answers recorded as either continuous variables (e.g., age) or categorical variables (e.g., marital status).

#### Adverse childhood experiences international questionnaire

The assessment of participants’ adverse childhood experiences (ACEs) was conducted using the Adverse Childhood Experiences International Questionnaire (ACE-IQ), formulated by the World Health Organization in 2020. The ACE-IQ is divided into 7 sections plus a demographic section. Each of the 7 sections consists of 1 to 8 statements for a total of 56 responses. Both the English (26) and Arabic (9) editions of the ACE-IQ were applied without modifications following extensive consultation with stakeholders on translation, back-translation, and cultural relevance. This tool is designed to evaluate the occurrence of ACEs and their potential link to risky behaviors in later life. This research employed the ACE-IQ to identify various childhood experiences encompassing physical, sexual, and emotional maltreatment, household dysfunction, violence from peers, the community, and larger groups, as well as neglect by parents or guardians. Responses were given based on participants’ retrospective perception, ranging from “Always” to “Never,” including “Refuse to answer,” with scores calculated per the ACE-IQ frequency scoring method.

#### Depression, anxiety, and stress scale

The Depression, Anxiety, and Stress Scale (DASS-21) (27) is a recognized tool for evaluating these three mental health aspects in adults, both clinical and non-clinical, via a singular, coherent, and structured 21-item questionnaire. Notably, this scale has been validated in Arabic (28). The scale asks participants to rate seven questions for depression, anxiety, and stress factors on the basis of a 0—never to 3—almost always Likert scale. Factors are then summed and multiplied by two in accordance with the standard instructions (27). Cronbach’s alpha assessments of internal consistency align with Antony et al. (29), exhibiting good-to-excellent internal consistency of 0.94 for depression, 0.87 for anxiety, and 0.91 for stress.

#### Health survey

Participants reported any formal health diagnoses and current lifestyle habits, responding to a series of questions with yes/no answers and the option to skip.

### Treatment of data

Data for each questionnaire were assessed for completeness, normality, skewness, and kurtosis, the size and random nature of the sample assured the independence of residual scores. Furthermore, the Mahalanobis, Cook’s, and leverage distances were examined to identify multivariate outlier cases within the sample, leading to a final sample for analyses of 697 respondents (30), from a total of 922 respondents who opened and engaged with the survey beyond the first page. The assumption of singularity was also met, highlighting that the explanatory variables did not exhibit perfect correlations and while there was some evidence of correlation across the independent variables, none were high and collinearity diagnostics were all within accepted limits. Residual output and scatterplots ensured the assumptions of normality, linearity, and homoscedasticity had also been met and autocorrelation was assured based on Durbin-Watson values between 1.5 and 2.5. All assumption testing and analyses were conducted using SPSS.

## Results

### Descriptive statistics

A series of hierarchical multiple regressions were conducted to examine the contributions of the predictor variables on the outcomes of depression, anxiety, and stress as measured by the DASS-21, using Bonferroni adjustment (see Table 1).

**Table 1:** Descriptive statistics, *N* = 697 listwise.

	*M*(SD)
Dependent variables	
DASS-21-Depression	7.01(9.31)
DASS-21-Anxiety	5.54(7.78)
DASS-21-Stress	8.14(9.39)
Predictors	
Average ACEs	1.28(1.69)
Covariates	
Age	38.39(9.73)

#### Depression

As illustrated in Table 2, block one of the multiple regression models seeks to understand and partial out the impact on depression of demographic factors (*R^2^*, *F*(3, 692) = 19.81, *p* < 0.001) before entering lifestyle behaviors (*R^2^*, *F*(8, 687) = 11.48, *p* < 0.001), followed by current health and mental health diagnoses (*R^2^*, *F*(16, 679) = 13.72, *p* < 0.001), finally entering ACEs in the final (fourth) regression block (*R^2^*, *F*(17, 678) = 17.11, *p* < 0.001). *R^2^* change data are available in Table 2. The findings indicate statistically significant accounts of variance in DASS-21 depression scores explained by factors present in each block of the analyses. Of note, when the impact of demographic factors (8%), lifetime risk behaviors (4%), and current medical and mental health diagnoses (12%) are considered, adverse childhood experiences continue to account for 6% of the variance in depression scores among this UAE community sample, which is to say that events which took place in childhood still explain greater than 5% of current levels of non-diagnosed, self-reported depression, based on DASS-D scores among this sample. Further examination of the final model demonstrates that age (B = −0.12 *t*(696) = −3.53, *p* < 0.001) and gender (B = 2.06 *t*(696) = −2.95, *p* < 0.005) significantly contribute to explained variance in depression screening values, highlighting that being younger and female are associated with higher depression scores in this sample. Use of prescription drugs (B = −2.79 *t*(696) = −3.52, *p* < 0.001), exercising at least three times a week (B = −1.67 *t*(696) = −2.69, *p* < 0.01), and having ever engaged in sexual behavior that one would self-define as “risky” (B = 3.46 *t*(696) = 2.08, *p* < 0.05) represented lifestyle factors associated with increases in depression scores. Meanwhile, the presence of a diagnosis of obesity (B = 2.92 *t*(696) = −3.21, *p* < 0.001), depression (B = 5.56 *t*(696) = 2.61, *p* < 0.01), and anxiety (B = 5.73 *t*(696) = 3.38, *p* < 0.001) represented the health and mental health diagnoses predictive of increases in depression screening values in the community.

**Table 2:** Coefficients for hierarchical regression of demographics, lifestyle, diagnosis, and ACEs on DASS-21 depression scores.

Model		Unstandardized coefficients		Standardized coefficients	t	Sig.	*R*	*R* ^2^	Adj *R*^2^	Δ*R*^2^
		B	Std. error	Beta						
1	(Constant)	7.75	1.91		4.04	<0.001	0.28	0.08***	0.08	–
	Age	−0.15	0.03	−0.16	−4.36	<0.001				
	Gender	3.01	0.72	0.16	4.16	<0.001				
	Emirati/expatriate	1.59	0.74	0.08	2.13	0.03				
2	(Constant)	9.31	2.08		4.48	<0.001	0.34	0.12***	0.11	0.04
	Age	−0.15	0.03	−0.15	−4.30	<0.001				
	Gender	2.75	0.77	0.14	3.56	<0.001				
	Emirati/expatriate	1.41	0.76	0.07	1.85	*0.06*				
	Ever smoked (cigarettes, shisha, cigars, or other types of tobacco)?	1.03	0.80	0.05	1.29	0.19				
	Consumed alcohol or other intoxicants on a regular basis?	−0.89	1.56	−0.02	−0.57	0.56				
	Use any prescription drugs beyond the recommended dosage?	−2.40	0.87	−0.10	−2.75	<0.01				
	Exercise on a regular basis?	−2.61	0.67	−0.14	−3.86	<0.001				
	Risky sexual behavior?	5.28	1.82	0.11	2.90	<0.005				
3	(Constant)	7.61	1.96		3.89	<0.001	0.49	0.24***	0.23	0.13
	Age	−0.12	0.03	−0.13	−3.61	<0.001				
	Gender	2.31	0.72	0.12	3.18	<0.005				
	Emirati/expatriate	0.40	0.73	0.02	0.55	0.58				
	Ever smoked (cigarettes, shisha, cigars, or other types of tobacco)?	1.00	0.75	0.05	1.33	0.18				
	Consumed alcohol or other intoxicants on a regular basis?	−1.03	1.46	−0.02	−0.70	0.48				
	Use any prescription drugs beyond the recommended dosage?	−3.06	0.82	−0.13	−3.72	<0.001				
	Exercise on a regular basis?	−1.79	0.64	−0.09	−2.77	<0.01				
	Risky sexual behavior?	5.58	1.70	0.11	3.28	<0.001				
	Diabetes	1.72	1.08	0.06	1.58	0.11				
	High Blood pressure	−0.18	1.05	−0.00	−0.17	0.86				
	Chronic respiratory problems	1.45	2.94	0.01	0.49	0.62				
	Obesity	3.47	0.94	0.13	3.68	<0.001				
	Cancer	3.26	4.13	0.02	0.78	0.43				
	Fibromyalgia/chronic pain or fatigue	5.21	5.83	0.03	0.89	0.37				
	Depression (diagnosed)	7.31	2.19	0.16	3.33	<0.001				
	Anxiety (diagnosed)	6.59	1.75	0.18	3.75	<0.001				
4	(Constant)	6.42	1.90		3.37	<0.001	0.55	0.30***	0.28	0.06
	Age	−0.12	0.03	−0.12	−3.53	<0.001				
	Gender	2.06	0.69	0.11	2.95	<0.005				
	Emirati/expatriate	0.01	0.70	0.00	0.01	0.98				
	Ever smoked (cigarettes, shisha, cigars, or other types of tobacco)?	0.87	0.72	0.04	1.20	0.22				
	Consumed alcohol or other intoxicants on a regular basis?	−1.02	1.41	−0.02	−0.72	0.47				
	Use any prescription drugs beyond the recommended dosage?	−2.79	0.79	−0.12	−3.52	<0.001				
	Exercise on a regular basis?	−1.67	0.62	−0.09	−2.69	<0.01				
	Risky sexual behavior?	3.46	1.66	0.07	2.08	<0.05				
	Diabetes	1.55	1.04	0.05	1.49	0.13				
	High blood pressure	−0.38	1.01	−0.01	−0.37	0.70				
	Chronic respiratory problems	1.13	2.83	0.01	0.39	0.69				
	Obesity	2.92	0.91	0.11	3.21	<0.001				
	Cancer	2.27	3.98	0.01	0.57	0.56				
	Fibromyalgia/chronic pain or fatigue	2.24	5.63	0.01	0.39	0.69				
	Depression (diagnosed)	5.56	2.12	0.12	2.61	<0.01				
	Anxiety (diagnosed)	5.73	1.69	0.15	3.38	<0.001				
	Overall ACEs	1.38	0.18	0.25	7.35	<0.001				

Dependent variable: DASS_D_Computed_final. For regression model *R*^2^. * *p* < 0.05; ** *p* < 0.01; *** *p* < 0.001. In all models, male is the reference category for gender, expatriate is the reference category for immigration status, and “No” is the reference category for all present/absent diagnostic and behavioral items.

#### Anxiety

Table 3 illustrates the same modeling in relation to the DASS anxiety subscale, where demographic factors (*R^2^*, *F*(3, 693) = 14.92, *p* < 0.001), lifestyle behaviors (*R^2^*, *F*(8, 688) = 9.05, *p* < 0.001), current health and mental health diagnoses (*R^2^*, *F*(16, 680) = 10.47, *p* < 0.001), and ACEs (*R^2^*, *F*(17, 679) = 13.19, *p* < 0.001) independently and collectively explain variance in levels of anxiety. Beyond demographic factors (which account for 6% of the variance), lifetime risk behaviors (4%), and current medical and mental health diagnoses (10%), adverse childhood experiences account for an additional 5% of the variance in anxiety scores among this UAE community sample, based on DASS-A scores. Further examination of the final model demonstrates that age (B = −0.10 *t*(696) = −3.42, *p* < 0.001) significantly contributes to explained variance in anxiety screening values, highlighting that being younger is associated with higher anxiety scores. Again, the use of prescription drugs (B = −2.38 *t*(696) = −3.46, *p* < 0.001), exercising at least three times a week (B = −1.16 *t*(696) = −2.15, *p* < 0.05), and having ever engaged in sexual behavior that you would self-define as “risky” (B = 3.09 *t*(696) = 2.14, *p* < 0.05) represented lifestyle factors associated with increases in depression scores. Meanwhile, the presence of a diagnosis of obesity (B = 2.92 *t*(696) = 3.21, *p* < 0.001), depression (B = 5.35 *t*(696) = 2.90, *p* < 0.005), and anxiety (B = 3.02 *t*(696) = 2.06, *p* < 0.05) represent the health and mental health diagnoses predictive of increases in depression screening values in the community.

**Table 3:** Coefficients for hierarchical regression of demographics, lifestyle, diagnosis, and ACEs on DASS-21 anxiety.

Model		Unstandardized coefficients		Standardized coefficients	T	Sig.	*R*	*R* ^2^	Adj *R*^2^	Δ*R*^2^
		B	Std. error	Beta						
1	(Constant)	6.60	1.61		4.09	<0.001	0.25	0.06***	0.06	-
	Age	−0.12	0.03	−0.16	−4.15	<0.001				
	Gender	2.21	0.61	0.14	3.63	<0.001				
	Emirati/expatriate	0.82	0.63	0.05	1.30	0.194				
2	(Constant)	8.74	1.76		4.97	<0.001	0.31	0.09***	0.09	0.04
	Age	−0.12	0.03	−0.15	−3.98	<0.001				
	Gender	1.62	0.65	0.10	2.48	0.014				
	Emirati/expatriate	0.45	0.65	0.03	0.69	0.490				
	Ever smoked (cigarettes, shisha, cigars, or other types of tobacco)?	−0.33	0.68	−0.02	−0.49	0.624				
	Consumed alcohol or other intoxicants on a regular basis?	−0.37	1.33	−0.01	−0.27	0.783				
	Use any prescription drugs beyond the recommended dosage?	−2.12	0.74	−0.11	−2.86	0.004				
	Exercise on a regular basis?	−1.85	0.57	−0.12	−3.23	0.001				
	By your own definition, have you ever engaged in risky sexual behavior?	4.57	1.54	0.11	2.97	0.003				
3	(Constant)	7.65	1.69		4.52	<0.001	0.45	0.25***	0.23	0.05
	Age	−0.11	0.03	−0.13	−3.49	<0.001				
	Gender	1.29	0.62	0.08	2.07	0.039				
	Emirati/expatriate	−0.20	0.63	−0.01	−0.31	0.755				
	Ever smoked (cigarettes, shisha, cigars, or other types of tobacco)?	−0.42	0.65	−0.03	−0.66	0.513				
	Consumed alcohol or other intoxicants on a regular basis?	−0.37	1.26	−0.01	−0.29	0.771				
	Use any prescription drugs beyond the recommended dosage?	−2.59	0.71	−0.14	−3.66	<0.001				
	Exercise on a regular basis?	−1.25	0.56	−0.08	−2.25	0.024				
	Risky sexual behavior?	4.77	1.46	0.12	3.26	0.001				
	Diabetes	0.44	0.93	0.02	0.47	0.636				
	High blood pressure	0.90	0.91	0.04	0.99	0.321				
	Chronic respiratory problems	1.54	2.53	0.02	0.61	0.542				
	Obesity	2.67	0.81	0.12	3.29	0.001				
	Cancer	3.16	3.56	0.03	0.89	0.376				
	Fibromyalgia/chronic pain or fatigue	9.28	5.02	0.06	1.85	0.065				
	Depression (diagnosed)	6.74	1.89	0.18	3.56	<0.001				
	Anxiety (diagnosed)	3.70	1.51	0.12	2.45	0.015				
4	(Constant)	6.68	1.65		4.06	<0.001	0.50	0.25***	0.23	0.05
	Age	−0.10	0.03	−0.13	−3.42	<0.001				
	Gender	1.09	0.61	0.07	1.81	0.071				
	Emirati/expatriate	−0.51	0.61	−0.03	−0.83	0.410				
	Ever smoked (cigarettes, shisha, cigars, or other types of tobacco)?	−0.53	0.63	−0.03	−0.84	0.402				
	Consumed alcohol or other intoxicants on a regular basis?	−0.36	1.22	−0.01	−0.29	0.771				
	Use any prescription drugs beyond the recommended dosage?	−2.38	0.69	−0.13	−3.46	<0.001				
	Exercise on a regular basis?	−1.16	0.54	−0.07	−2.15	0.032				
	Risky sexual behavior?	3.09	1.44	0.08	2.14	0.032				
	Diabetes	0.31	0.91	0.01	0.35	0.729				
	High Blood pressure	0.75	0.88	0.03	0.85	0.397				
	Chronic respiratory problems	1.28	2.45	0.02	0.52	0.601				
	Obesity	2.24	0.79	0.10	2.84	0.005				
	Cancer	2.38	3.45	0.02	0.69	0.491				
	Fibromyalgia/chronic pain or fatigue	6.92	4.88	0.05	1.42	0.157				
	Depression (diagnosed)	5.35	1.84	0.14	2.90	0.004				
	Anxiety(diagnosed)	3.02	1.47	0.10	2.06	0.040				
	Overall ACEs	1.10	0.16	0.24	6.76	<0.001				

Dependent variable: DASS_A_Computed_final. For regression model *R*^2^. * *p* < 0.05; ** *p* < 0.01; *** *p* < 0.001. In all models, male is the reference category for gender, expatriate is the reference category for immigration status, and “No” is the reference category for all present/absent diagnostic and behavioral items.

#### Stress

Table 4 illustrates the same modeling in relation to the DASS stress subscale, where demographic factors (*R^2^*, *F*(3, 696) = 26.57, *p* < 0.001), lifestyle behaviors (*R^2^*, *F*(8, 688) = 14.33, *p* < 0.001), current health and mental health diagnoses (*R^2^*, *F*(16, 680) = 14.59, *p* < 0.001), and ACEs (*R^2^*, *F*(17, 679) = 18.21, *p* < 0.001) independently and collectively explain variance in levels of stress, with demographic factors (10%), lifetime risk behaviors (4%), current medical and mental health diagnoses (11%), and adverse childhood experience accounting for 6% of the variance in stress scores among this UAE community sample, which is to say that events which took place in childhood still explain almost 6% of the variance in current levels of stress in adults, based on DASS-S scores among this sample. Further examination of the final model demonstrates that age (B = −0.10 *t*(696) = −2.89, *p* < 0.005) and gender (B = 3.18 *t*(696) = 4.55, *p* < 0.001) significantly contribute to explained variance in anxiety screening values, highlighting that being younger and female is associated with higher stress scores. Again, the use of prescription drugs (B = −2.30 *t*(696) = −2.90, *p* < 0.005) and exercising at least three times a week (B = −1.91 *t*(696) = −3.08, *p* < 0.005) represented lifestyle factors associated with increases in stress scores. Meanwhile, the presence of a diagnosis of obesity (B = 3.12 *t*(696) = 3.42, *p* < 0.001), depression (B = 5.20 *t*(696) = 2.45, *p* < 0.05), and anxiety (B = 5.29 *t*(696) = 3.12, *p* < 0.005) represent the health and mental health diagnoses predictive of increases in stress screening values.

**Table 4:** Coefficients for hierarchical regression of demographics, lifestyle, diagnosis, and ACEs on DASS-21 stress.

Model		Unstandardized coefficients		Standardized coefficients	T	Sig.	*R*	*R* ^2^	Adj *R*^2^	Δ*R*^2^
		B	Std. error	Beta						
1	(Constant)	6.07	1.90		3.19	0.001	0.32	0.10***	0.10	–
	Age	−0.13	0.04	−0.13	−3.65	<0.001				
	Gender	4.09	0.72	0.22	5.68	<0.001				
	Emirati/expatriate	2.09	0.74	0.11	2.81	0.005				
2	(Constant)	7.61	2.07		3.68	<0.001	0.38	0.14***	0.13	0.04
	Age	−0.13	0.04	−0.14	−3.72	<0.001				
	Gender	3.87	0.77	0.21	5.04	<0.001				
	Emirati/expatriate	2.10	0.76	0.11	2.77	0.006				
	Ever smoked (cigarettes, shisha, cigars, or other types of tobacco)?	0.98	0.80	0.05	1.23	0.220				
	Consumed alcohol or other intoxicants on a regular basis?	1.40	1.56	0.03	0.89	0.371				
	Use any prescription drugs beyond the recommended dosage?	−1.99	0.87	−0.09	−2.28	0.023				
	Exercise on a regular basis?	−2.85	0.68	−0.15	−4.22	<0.001				
	Risky sexual behavior?	4.65	1.81	0.10	2.57	0.010				
3	(Constant)	5.97	1.97		3.04	0.002	0.50	0.26***	0.24	0.11
	Age	−0.11	0.04	−0.11	−2.97	0.003				
	Gender	3.43	0.73	0.18	4.73	<0.001				
	Emirati/expatriate	1.24	0.73	0.06	1.69	0.092				
	Ever smoked (cigarettes, shisha, cigars, or other types of tobacco)?	0.88	0.75	0.05	1.17	0.242				
	Consumed alcohol or other intoxicants on a regular basis?	1.26	1.47	0.03	0.86	0.391				
	Use any prescription drugs beyond the recommended dosage?	−2.57	0.82	−0.12	−3.13	0.002				
	Exercise on a regular basis?	−2.04	0.65	−0.11	−3.16	0.002				
	Risky sexual behavior?	4.85	1.70	0.10	2.85	0.005				
	Diabetes	0.42	1.09	0.01	0.39	0.699				
	High blood pressure	0.46	1.06	0.02	0.43	0.667				
	Chronic respiratory problems	−1.15	2.94	−0.01	−0.39	0.696				
	Obesity	3.67	0.94	0.14	3.89	<0.001				
	Cancer	5.24	4.14	0.04	1.27	0.206				
	Fibromyalgia/chronic pain or fatigue	4.48	5.84	0.03	0.77	0.443				
	Depression (diagnosed)	6.98	2.20	0.15	3.18	0.002				
	Anxiety (diagnosed)	6.17	1.76	0.17	3.51	<0.001				
4	(Constant)	4.72	1.90		2.49	0.013	0.56	0.31***	0.29	0.06
	Age	−0.10	0.03	−0.10	−2.89	0.004				
	Gender	3.18	0.70	0.17	4.55	<0.001				
	Emirati/expatriate	0.84	0.71	0.04	1.19	0.236				
	Ever smoked (cigarettes, shisha, cigars, or other types of tobacco)?	0.75	0.72	0.04	1.04	0.300				
	Consumed alcohol or other intoxicants on a regular basis?	1.28	1.41	0.03	0.90	0.367				
	Use any prescription drugs beyond the recommended dosage?	−2.30	0.79	−0.10	−2.90	0.004				
	Exercise on a regular basis?	−1.91	0.62	−0.10	−3.08	0.002				
	Risky sexual behavior?	2.68	1.66	0.06	1.61	0.108				
	Diabetes	0.26	1.04	0.01	0.24	0.807				
	High Blood pressure	0.26	1.02	0.01	0.25	0.802				
	Chronic respiratory problems	−1.48	2.83	−0.02	−0.53	0.600				
	Obesity	3.12	0.91	0.12	3.42	<0.001				
	Cancer	4.24	3.98	0.03	1.06	0.288				
	Fibromyalgia/chronic pain or fatigue	1.44	5.63	0.01	0.26	0.798				
	Depression (diagnosed)	5.20	2.13	0.11	2.45	0.015				
	Anxiety (diagnosed)	5.29	1.69	0.15	3.12	0.002				
	Overall ACEs	1.42	0.19	0.26	7.54	<0.001				

Dependent variable: DASS_S_Computed_final. For regression model *R*^2^. * *p* < 0.05; ** *p* < 0.01; *** *p* < 0.001. In all models, male is the reference category for gender, expatriate is the reference category for immigration status, and “No” is the reference category for all present/absent diagnostic and behavioral items.

## Discussion

This study underscores the importance of ACEs on adult health and wellbeing, particularly for the UAE population where there appear to be a relatively low number of average ACEs. This supports the recent findings of Long et al. (14), and is indeed, lower than those exhibited in Saudi Arabia, serving as a regional comparator (10). Key to these conclusions is the variance accounted for by ACEs in depression (6%), anxiety (5%), and stress scores (6%), controlling for covariates. Importantly, increases in age, use of prescription drugs, and exercising three or more times per week all appear to be playing a protective role. Meanwhile, greater levels of depression, anxiety, and stress are associated with being female (in the cases of depression and stress), having previously engaged in risky sexual behaviors (in the cases of depression and stress), and having received formal diagnoses of obesity, depression, and anxiety. These results underline the persistent influence of early life stressors on mental health into adulthood as well as possible protective factors for this population, aligning with global research that highlights the enduring impact of ACEs on psychological wellbeing and disease (20).

Given these findings alongside emerging support and guidance for research and action at the public health and policy levels (21, 31–33), several broader implications emerge, which are aimed at mitigating the long-term impacts of ACEs on mental health within the UAE. First, early intervention and prevention strategies are critical. Screening for ACEs in settings frequented by children and adolescents, such as schools and healthcare facilities, can facilitate early identification and support (34). Furthermore, integrating mental health services within primary healthcare settings can ensure that individuals with a history of ACEs receive comprehensive care, addressing both physical and psychological needs (26). Such approaches serve to benefit from system-level approaches, prioritized policy and resource allocation, and approaches that are grounded in an appropriate philosophy, such as trauma-informed care (34–38).

Public awareness campaigns can play a vital role in educating the community about the significance of ACEs and the importance of seeking early intervention. Such campaigns can reduce stigma and promote a culture of support and understanding (35). Additionally, training for professionals who work with children, such as teachers, healthcare providers, and social workers, is essential (36). This training should focus on recognizing the signs of adversity, understanding the potential impacts on long-term health, and providing appropriate interventions.

Supportive policies that offer resources to families at risk of ACEs are also crucial. Access to counseling services, financial assistance, and parenting programs can help prevent the occurrence of ACEs and provide support to those affected (37). Finally, ongoing research and monitoring are necessary to understand the prevalence and impacts of ACEs fully, particularly in vulnerable and understudied populations. This includes longitudinal studies to track the long-term health outcomes of individuals with ACEs, the development of culturally appropriate interventions, evaluation of these interventions, and assessment of policy and community-level impact (38, 39).

### Limitations

While informative and in its contributions to regional and contextual knowledge, several limitations are observed in the implementation of this study. Despite their benefit in partially addressing the challenges of stigmatization impacting reporting in the region (15), self-report measures are susceptible to bias. Significantly, retrospective ACEs may be subject to the impact of recall bias (40, 41). However, recent findings do support that the subjective experiences associated with memories of childhood trauma may be more deleterious to mental health than the events themselves (42). Additionally, the use of self-report measures for predictors and outcomes creates shared method variance. The cross-sectional nature of the design does limit our ability to draw causal conclusions about the role ACEs play in psychological suffering and distress. Finally, the lack of a complex sample clustering method may limit the true representativeness of the sample.

### Future research

Future research developments in the study of the long-term impact of ACEs and later vulnerabilities to psychological distress should prioritize prospective approaches to the study of ACEs (21). The potential ability to control for genetic and early environmental risk factors could enhance the explanatory power of the role of ACEs themselves. Further deconstructing type and frequency or patterns of exposure to different ACEs may help to differentiate phenotypic vulnerabilities to psychological distress.

## Conclusion

In conclusion, addressing ACEs through a comprehensive, multi-sectoral approach can significantly improve mental health outcomes for individuals in the UAE. By implementing targeted policies and interventions, it is possible to mitigate the effects of early adversity, enhancing both individual and societal wellbeing.

## Data availability statement

The raw data supporting the conclusions of this article may be made available by the authors, without undue reservation.

## Ethics statement

The studies involving humans were approved by Georgetown University Medical Center (STUDY00004558). The studies were conducted in accordance with the local legislation and institutional requirements. The participants provided their written informed consent to participate in this study.

## Author contributions

AM: Conceptualization, Data curation, Formal analysis, Investigation, Methodology, Project administration, Validation, Writing – original draft, Writing – review & editing. DE: Data curation, Formal analysis, Visualization, Writing – original draft, Writing – review & editing. IE: Conceptualization, Data curation, Formal analysis, Investigation, Methodology, Project administration, Visualization, Writing – original draft, Writing – review & editing. NH: Funding acquisition, Resources, Supervision, Validation, Writing – review & editing. TL: Conceptualization, Funding acquisition, Project administration, Resources, Supervision, Writing – review & editing. ZI-A: Writing – review & editing. CA: Funding acquisition, Project administration, Resources, Supervision, Writing – review & editing.
